# Deposition of Inhaled Levofloxacin in Cystic Fibrosis Lungs Assessed by Functional Respiratory Imaging

**DOI:** 10.3390/pharmaceutics13122051

**Published:** 2021-12-01

**Authors:** Carsten Schwarz, Claudio Procaccianti, Benjamin Mignot, Hosein Sadafi, Nicolas Schwenck, Xabier Murgia, Federico Bianco

**Affiliations:** 1 CF Center, 14467 Potsdam, Germany; pddrschwarz@gmail.com; 2 Global Medical Affairs, Chiesi Farmaceutici S.p.A., 43122 Parma, Italy; c.procaccianti@chiesi.com; 3 FLUIDDA NV, 2550 Kontich, Belgium; Benjamin.Mignot@fluidda.com (B.M.); hosein.sadafi@fluidda.com (H.S.); 4 PARI Pharma GmbH, 82319 Starnberg, Germany; Nicolas.Schwenck@pari.com; 5 Scientific Consultancy, 48640 Bilbao, Spain; xabi_murgia@hotmail.com

**Keywords:** inhaled levofloxacin, *Pseudomonas aeruginosa*, cystic fibrosis, vibrating-membrane nebuliser, functional respiratory imaging, lung deposition

## Abstract

Pulmonary infections caused by *Pseudomonas aeruginosa* (PA) represent the leading cause of pulmonary morbidity in adults with cystic fibrosis (CF). In addition to tobramycin, colistin, and aztreonam, levofloxacin has been approved in Europe to treat PA infections. Nevertheless, no lung deposition data on inhaled levofloxacin are yet available. We conducted a Functional Respiratory Imaging (FRI) study to predict the lung deposition of levofloxacin in the lungs of patients with CF. Three-dimensional airway models were digitally reconstructed from twenty high-resolution computed tomography scans obtained from historical patients’ records. Levofloxacin aerosols generated with the corresponding approved nebuliser were characterised according to pharmacopeia. The obtained data were used to inform a computational fluid dynamics simulation of levofloxacin lung deposition using breathing patterns averaged from actual CF patients’ spirometry data. Levofloxacin deposition in the lung periphery was significantly reduced by breathing patterns with low inspiratory times and high inspiratory flow rates. The intrathoracic levofloxacin deposition percentages for moderate and mild CF lungs were, respectively, 37.0% ± 13.6 and 39.5% ± 12.9 of the nominal dose. A significant albeit modest correlation was found between the central-to-peripheral deposition (C/P) ratio of levofloxacin and FEV_1_. FRI analysis also detected structural differences between mild and moderate CF airways. FRI revealed a significant intrathoracic deposition of levofloxacin aerosols, which distributed preferentially to the lower lung lobes, with an influence of the deterioration of FEV_1_ on the C/P ratio. The three-dimensional rendering of CF airways also detected structural differences between the airways of patients with mild and moderate CF.

## 1. Introduction

Pulmonary infections caused by *Pseudomonas aeruginosa* (PA) represent the leading cause of pulmonary morbidity and mortality in adults with cystic fibrosis (CF) [1]. Chronic lung infections are associated with a gradual deterioration of lung function, which is denoted clinically by a progressive decline of the forced expiratory volume in one second (FEV_1_) [2] and structural abnormalities on computed tomography (CT) scans [3].

Inhaled antipseudomonal antibiotics administered early after bacterial colonisation can eradicate PA from the CF lungs [4]. In addition, the topical delivery of antibiotics reduces the intrapulmonary bacterial load, the number of exacerbations, and improves lung function [5]. Despite the clear benefits of inhaled antibiotics in CF, there are just a few treatment options approved, namely, tobramycin, aztreonam, colistin, and, more recently, levofloxacin [5]. Interestingly, they belong to different antibiotic subgroups with diverse mechanisms of action, providing alternative treatment options for PA strains that may have developed resistance to certain antibiotic types.

The lung deposition and the pulmonary distribution of inhaled antibiotics must be sufficient to achieve local concentrations above the minimum inhibitory concentration (MIC) that will further enable bacterial killing. Nevertheless, lung deposition depends on several factors such as the aerosol characteristics, breathing pattern, and the structure of the airway tree. Considering that the latter factors may be markedly affected by CF progression, a high patient-to-patient variability can be expected in terms of lung deposition, which can be inferred to some extent from the wide ranges of antibiotic sputum concentrations reported in clinical studies of CF [6]. Average estimations of the delivered lung dose are required for the regulatory approval of inhaled drug formulations. They are usually determined using straightforward in vitro set-ups [7], which, unfortunately, neither provide data on lung distribution nor consider the respiratory pattern or the CF airways’ pathophysiological features.

Functional Respiratory Imaging (FRI) represents an appealing alternative to scintigraphy studies to investigate the lung deposition of aerosols in human airways. FRI uses metadata from high-resolution CT scans to render three-dimensional models of the airway tree, which are then used to run computational fluid dynamics (CFD) simulations that can accurately predict the lung deposition of well-characterised aerosol plumes [8,9]. Interestingly, FRI does not require drug labelling and can be implemented using retrospective CT scans. Therefore, FRI can be used to simultaneously scrutinise morphological features of disease progression and investigate factors that affect lung deposition.

The present study was designed to investigate the lung deposition of levofloxacin aerosols using FRI within the airways of mild and moderate CF patients’ airways, which were selected as the most representative population for maintenance therapy with inhaled antibiotics. For that purpose, we first performed a characterisation of levofloxacin aerosols generated with the officially approved nebuliser, which was used in combination with breathing patterns averaged from spirometry data of actual CF patients to determine the lung deposition and the pulmonary distribution of a clinical dose of inhaled levofloxacin.

## 2. Materials and Methods

### 2.1. Aerosol Characterisation

Levofloxacin (Quinsair, Chiesi Farmaceutici, Parma, Italy) aerosols generated with the approved nebuliser Zirela (PARI Pharma GmbH, Starnberg, Germany) were characterised according to the Pharmacopeia [7]. Zirela is a vibrating-membrane nebuliser based on eFlow Technology, which is operated by the eBase controller (PARI Pharma GmbH, Starnberg, Germany).

The delivered dose and the levofloxacin delivery rate were determined in vitro using a set-up that consisted of a Zirela nebuliser, two drug collection filters (PARI filter pads PARI GmbH, Starnberg, Germany) respectively placed immediately after the nebuliser and at the expiratory limb, and a breath simulator (Compas 2, PARI Pharma, Starnberg, Germany) programmed with a sinusoidal waveform to deliver a tidal volume (*V*_T_) of 500 mL at a rate of 15 cycles/minute and an inspiration/exhalation (I:E) ratio of 1:1. A clinical dose of 240 mg of levofloxacin at a 100 mg/mL concentration was delivered in each experiment.

The aerodynamic particle size distribution (APSD) was determined using the Next Generation Impactor (NGI, Copley Scientific, Nottingham, UK) [10]. The experiments were conducted at 23 °C and 50% relative humidity conditions and inspiratory flow of 15 L/min. After the nebulisation of a clinical levofloxacin dose, the impactor’s stages were rinsed with the appropriate solvent for levofloxacin analysis. Additionally, the amount of levofloxacin remaining in the nebuliser, in the artificial throat of the NGI, and the micro-orifice collector (MOC) filter were determined by high-performance liquid chromatography (HPLC, Supplemental Method 1). The mass median aerodynamic diameter (MMAD), the geometric standard deviation (GSD), and the fine particle fraction (FPF) were determined. All experiments were repeated ten times, each with an independent nebuliser.

### 2.2. Human-Based Cystic Fibrosis Airway Modelling

Retrospective high-resolution CT scans from 20 historical adult patients with CF were obtained from the FLUIDDA database (informed consent was obtained from each patient, and ethical approval was granted by the Institutional Review Board [IRB] of the University Hospital in Antwerp, Belgium; file number: B300201731264; approval date 25/08/2017). Segmentation and 3D model operations were performed in commercially available validated software packages (Mimics 20.0 and 3-Matic 12.0, Materialise nv, Belgium). The airway tree (i.e., intraluminal air) could be segmented down to bronchi with a diameter of about 1–2 mm. Beyond this point, the CT resolution is insufficient to distinguish alveolar and intraluminal air. Segmentation was semi-automatic, with airways then manually checked and missing branches added. A typical airway model includes 5–10 generations, depending mainly on the disease state of the individual patient. The device geometry was reverse-engineered into a 3D computer-aided design model, and then virtually coupled to the patients’ airway models.

Additionally, data regarding patients’ sex, age, height, FEV_1_, and FEV_1_ (%pred) were retrieved (Table 1, Supplemental Table S1). CF stage was classified as mild if the FEV_1_ (%pred) > 70% or as moderate if the FEV_1_ (%pred) was in the 40–70% range, according to the guidelines of the Cystic Fibrosis Foundation Patient Registry [11]. Seven patients were included in the mild CF group and 13 were included in the moderate CF group. There were no significant differences between groups regarding sex, age, or height.

### 2.3. Computational Fluid Dynamic (CFD) Simulation

The MMAD, GSD, FPF, and the delivered dose obtained from in vitro experiments were used as input parameters for the CFD simulations in the CF airways. The surface meshing strategy and the boundary conditions for the simulation have been described elsewhere [8,9]. The patient-specific models included the extrathoracic region, comprising the mouth and upper airways, and the intrathoracic airways, which were divided into (1) central airways, from the start of the trachea and including all the airways visible on a high-resolution CT scan; and (2) the peripheral airways. The intrathoracic deposition was divided into regional lobar areas: right upper lobe, right middle lobe, right lower lobe, left upper lobe, and left lower lobe. Moreover, the intrathoracic levofloxacin deposition was also classified as central deposition if aerosol particles were deposited in the airways that were well defined in the reconstructed CT-based three-dimensional model, or as peripheral deposition if the particles deposited beyond the airways visible in the three-dimensional model.

Three different sinusoidal breathing patterns were used: the first breathing pattern consisted of a *V*_T_ of 0.5 L, rate of 14 cycles/min, I:E 1:1.5, and a mean flow rate of 17.5 L/min, and we refer to it as the “regular breathing” pattern; it approaches the pattern described by the pharmacopoeia [7], although with a slight difference in terms of respiratory rate (14 instead of 15 bpm) and I:E (1:1.5 instead of 1:1). Two additional breathing patterns averaged from two groups of patients with mild and moderate CF were retrieved from the PARI database (from patients recruited from the CF clinics of the Hospital for Sick Children and St. Michael’s Hospital in Toronto; all patients signed informed consent and the study was approved by the IRB of both institutions, Supplemental Table S2):

(1) Mild CF breathing pattern: *V*_T_ of 0.759 L, rate of 21 cycles/min, I:E 1:2.3, and a mean flow rate of 46.5 L/min; and
(2) Moderate CF breathing pattern: *V*_T_ of 0.608 L, rate of 23 cycles/min, I:E 1:2.14, and a mean flow rate of 50 L/min.

### 2.4. Statistical Analysis

The data are presented as mean ± standard deviation (SD). Unless otherwise stated, one-way ANOVA was used to analyse the datasets (SPSS Statistics software version 23, IBM Corp, Armonk, NY, USA). The correlation analysis was performed using Pearson’s correlation test. A *p* < 0.05 was accepted to determine a statistical significance.

## 3. Results and Discussion

Inhaled antibiotics have dramatically improved the management of PA infections in CF patients. Unfortunately, long-term antibiotic treatment generates evolutionary pressure over the highly adaptable PA, ultimately developing resistance and persisting in the host’s lungs. In this scenario, the approval of alternative inhaled antibiotics is indispensable to provide complementary treatment options for PA strains displaying resistance to certain antibiotic types.

In addition to tobramycin, colistin, and aztreonam, inhaled levofloxacin has been approved in Europe to treat PA infections in patients with CF [12]. Levofloxacin is a fluoroquinolone with a broad spectrum for Gram-negative bacteria, which inhibits the bacterial DNA gyrase, precluding the synthesis of functional bacterial DNA [13]. Clinical trials have demonstrated that inhaled levofloxacin significantly reduces the sputum PA load and improves the FEV_1_ (%pred) compared with placebo treatment [14,15], and they confirmed the non-inferiority of levofloxacin compared with tobramycin solution [16]. In a real-world setting study, the new start of inhaled levofloxacin in patients with CF led to a significant improvement in FEV_1_ (%pred) and a significant reduction of exacerbation rate [17]. Levofloxacin is very active against PA isolates growing in biofilms in vitro, and it has been shown to be more potent than both the aminoglycosides tobramycin and amikacin, and also aztreonam [6,18]. Nevertheless, the efficacy of inhaled levofloxacin does not only depend on the intrinsic characteristics of the antibiotic but is also contingent on the overall lung deposition and its intrapulmonary distribution. Motivated by the lack of data on the lung deposition of levofloxacin, we designed an FRI-based study to shed light on the regional distribution of levofloxacin in CF lungs.

### 3.1. Aerosol Characterisation

Inhaled levofloxacin therapy has been approved to be delivered specifically in combination with the Zirela nebuliser. Therefore, we first conducted a thorough characterisation of levofloxacin aerosols according to international regulatory standards [7]. The mean delivered dose of levofloxacin determined from breath simulation experiments (i.e., levofloxacin mass collected in the inspiratory filter) was 164 ± 10 mg, representing 68% of the dose placed in the nebuliser chamber. The mean levofloxacin delivery rate was 24 ± 5 mg/min. Interestingly, the mean residual amount of levofloxacin remaining in the vibrating-membrane nebuliser was just 10% ± 3 of the nominal dose, which is low compared to jet nebulisers used in CF, such as the PARI LC Plus nebuliser in which the residual volume may account for up to 43.5% of the nominal dose [19].

**Figure 1:**
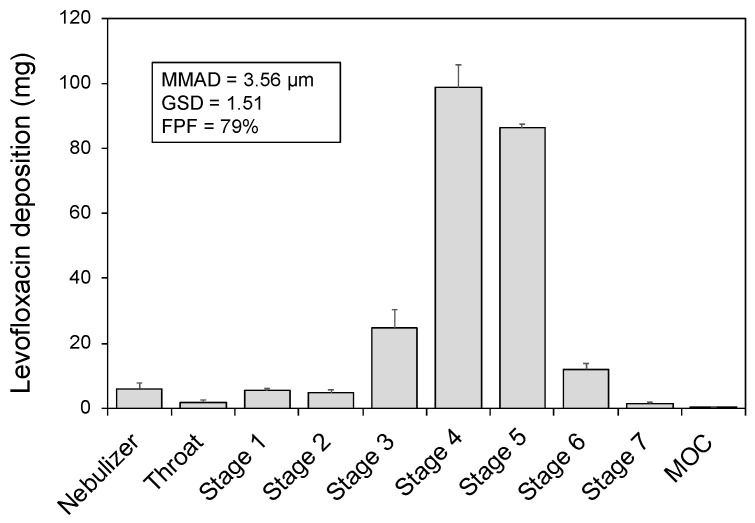
Levofloxacin deposition in components of the in vitro set-up during next-generation cascade impactor experiments. MMAD, mass median aerodynamic diameter; GSD, geometric standard deviation; FPF, fine particle fraction.

Figure 1 displays the outcome of the APSD experiments performed with the NGI and shows the amount of levofloxacin deposited in each set-up component. The highest amounts of levofloxacin aerosols deposited onto stages 4 and 5, respectively corresponding with cut-off diameters of 3.30 and 2.08 µm. Accordingly, the mean MMAD was 3.56 ± 0.12 µm (GSD of 1.51 ± 0.04) and the FPF was 79% ± 3.

The FPF is of high relevance since it expresses the percentage of particles in the aerosol plume with aerodynamic diameter below 5 µm, which are more likely to achieve a peripheral deposition [20]. Combining the data from breath simulation experiments with the FPF obtained from the NGI experiments, a mean respirable dose of 130 mg was determined, representing approximately 54% of the nominal dose.

### 3.2. Morphometric Analysis of CF Airways

**Figure 2:**
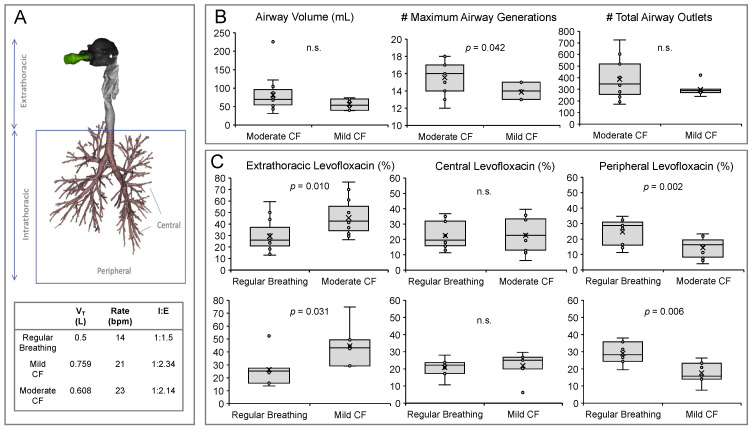
(**A**) Representative three-dimensional rendering from a high-resolution computed tomography scan from a cystic fibrosis patient. Different aerosol deposition regions are described in the figure; below, a table summarises the three different breathing patterns used in the study. (**B**) Comparison of the airway volume, maximum airway generation, and total airway outlets determined in mild and moderate cystic fibrosis airways; n.s., non-significant. (**C**) Top row: Extrathoracic, central airway, and peripheral lung deposition of levofloxacin achieved with either the “regular breathing” pattern or the moderate CF breathing pattern in thirteen patients with moderate cystic fibrosis. Bottom row: Extrathoracic, central airway, and peripheral lung deposition of levofloxacin achieved with either the “regular breathing” pattern or the mild CF breathing pattern in seven patients with mild cystic fibrosis; n.s., non-significant.

CT scans are useful in CF for routine monitoring of disease progression, since they are more sensitive to early structural changes of the airways than pulmonary function testing [3]. Beyond qualitative findings, the potential of CT findings as a surrogate outcome for clinical trials has also been proposed through the development of different CT scoring systems, including automated and non-automated systems [21]. In this work, we used high-resolution CT scans from historical CF patient records to reconstruct three-dimensional models of the airways that in combination with the aerosol characterisation data were used to inform the CFD simulation of levofloxacin lung deposition. The reconstructed CF lungs were first divided into extrathoracic and intrathoracic deposition areas, and the intrathoracic area was further segregated into central and peripheral deposition zones (Figure 2A). We determined a few structural features of the CF airways, including the total airway volume, the maximum number of generations, and the total airway outlets of patients with mild and moderate CF. We found a significantly higher number of detected maximum airway generations in the airways of patients with a moderate CF as well as a non-significant trend towards a higher airway volume and higher total airway outlets (Figure 2B); since there were neither differences in age nor height between the mild and moderate CF patient groups, we speculate that these changes can be attributed to structural changes associated with CF progression such as bronchiectasis, bronchus obstructions, and trapped air [22]. Complementary to already existing CT scoring systems, these findings indicate that FRI could eventually be used as an unbiased semi-quantitative method to determine the structural and morphological features of CF airways.

### 3.3. Comparison of Levofloxacin Deposition with Regular or CF Breathing Patterns

We first applied FRI to compare the lung deposition of levofloxacin achieved with the “regular breathing” pattern with the moderate CF breathing pattern in thirteen patients (Figure 2C, top row), and with the mild breathing pattern in the remaining seven patients (Figure 2C, bottom row). Both comparisons showed a similar trend: irrespective of mild or moderate, both CF breathing patterns were associated with a significantly higher mean extrathoracic deposition than the “regular breathing” pattern, indicating less intrapulmonary lung bioavailability of levofloxacin for the CF breathing patterns. Significant differences were also noted in the peripheral deposition, with both CF breathing patterns showing a significantly lower peripheral deposition. The observed significant differences may be explained by the shorter inspiratory time and the higher *V*_T_ and respiratory rate of the CF breathing patterns, which show a steeper slope of the flow profile and a higher peak inspiratory flow that may eventually promote the impaction of levofloxacin in the upper airways (Supplemental Figure S1). These observations highlight the relevance of instructing CF patients on acquiring a good inhalation technique in order to maximise the intrapulmonary dose of antibiotics [23].

### 3.4. Comparison of Levofloxacin Deposition on Mild or Moderate CF Airways

**Figure 3:**
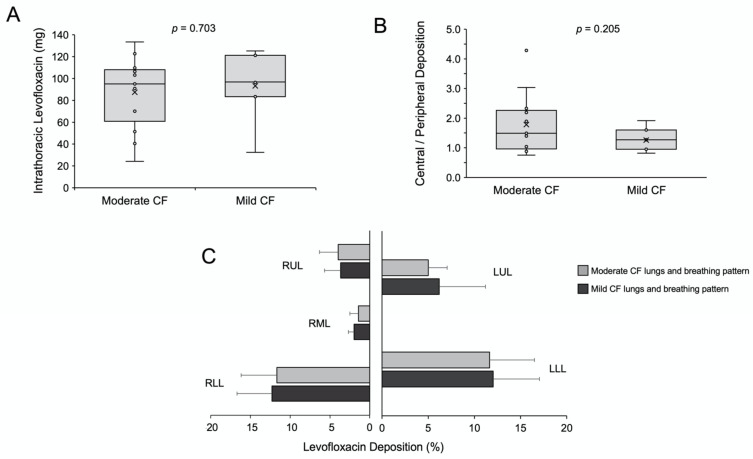
(**A**) Mean intrathoracic deposition in moderate and mild cystic fibrosis lungs. (**B**) Central to peripheral deposition (C/P) ratio in moderate and mild cystic fibrosis lungs. (**C**) Regional levofloxacin deposition in the moderate (light grey, *n* = 13) and mild (dark grey, *n* = 7) cystic fibrosis lungs. RUL, right upper lobe; RML, right middle lobe; RLL, right lower lobe; LUL, left upper lobe; LLL, left lower lobe.

Furthermore, we compared the overall and the regional lung deposition of levofloxacin in patients with moderate and mild CF. The mean intrathoracic deposition in the moderate CF group was 87.5 mg (range 24–133 mg), and it was slightly but not significantly lower compared with the mild CF group: 93.1 mg (range 32–125 mg, Figure 3A); these levofloxacin amounts respectively account for mean lung deposition percentages of 37.0% ± 13.6 and 39.5% ± 12.9 of the nominal dose. These values are higher compared with scintigraphy data from healthy volunteers and CF patients who inhaled tobramycin in solution either using the LC plus jet nebuliser or the eFlow rapid vibrating-membrane nebuliser (both from PARI, Starnberg, Germany), who achieved median lung deposition rates below 15% [19,24]. The lung deposition of levofloxacin with Zirela was also higher compared with the scintigraphy deposition data reported for colistimethate sodium (colomycin 80 mg) in patients with CF, which was just 5.9% of the nominal dose [25]. These differences may be partially explained by the patient variability across the studies, but also by differences in the nebuliser type and the aerosol characteristics [24].

**Figure 4:**
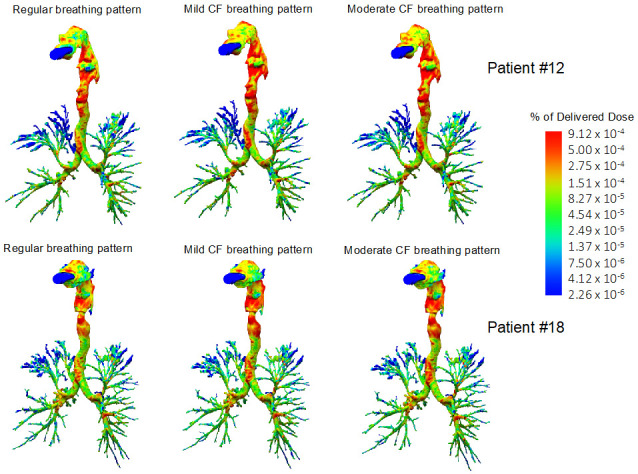
Three-dimensional reconstruction of the airways and levofloxacin aerosol deposition in two representative cystic fibrosis (CF) patients (Patient #12, moderate CF, top row; Patient #18, mild CF, bottom row). Computational fluid dynamics simulations showed a higher extrathoracic levofloxacin deposition with mild and moderate CF breathing patterns compared with the “regular breathing” pattern. Levofloxacin aerosols distributed preferentially to the lower lung lobes, irrespective of the breathing pattern.

Although the peripheral deposition was slightly higher in the mild CF group, there were no significant differences for the central-to-peripheral (C/P) ratio or the regional deposition across the different lung lobes (Figure 3B). However, levofloxacin aerosols distributed preferentially to the lower lung lobes (Figure 3C, Figure S2 and Figure 4). The lack of differences between mild and moderate CF groups in peripheral deposition can be best explained by the fact that the differences between both groups’ breathing patterns are not so evident. The inclusion of just two averaged representative CF breathing patterns is a limitation of the study. In the future, breathing pattern recordings combined with high-resolution CT scans may provide a personalised estimation of lung deposition for each tested patient.

### 3.5. Correlation between Levofloxacin Deposition and FEV_1_ and FEV_1_ (%pred)

**Figure 5:**
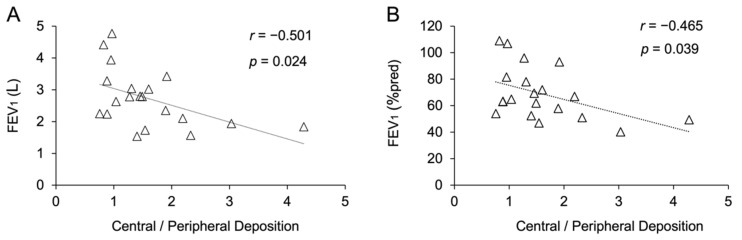
(**A**) Correlation between the forced expiratory volume in 1 s (FEV_1_) and the central-to-peripheral deposition ratio. (**B**) Correlation between the FEV_1_ compared to the FEV_1_ of the reference population (FEV_1_ (%pred)) and the central-to-peripheral deposition ratio.

Changes in FEV_1_ and FEV_1_ (%pred) remain key functional outcomes to monitor and phenotype patients with CF [26,27]. Nevertheless, FEV_1_ and FEV_1_ (%pred) changes do not remain constant across all ages or all severity stages [28]. We performed a correlation analysis between the outcome values obtained from the FRI analysis and FEV_1_ and FEV_1_ (%pred); we did not find any significant correlation between FEV_1_ or FEV_1_ (%pred) and the airway volume, maximum airway generation, total airway outlets, exhaled levofloxacin, or extrathoracic, intrathoracic, central, and peripheral levofloxacin deposition. However, significant correlations between the central-to-peripheral deposition ratio and both FEV_1_ (%pred) and FEV_1_ were found (Figure 5A,B). Unexpectedly, the significant difference observed between mild and moderate CF patients in the maximum number of airway generations defined in the three-dimensional model was not correlated with FEV_1_ (%pred) or FEV_1_, which encourages the use of pulmonary imaging as a complement to functional testing for the phenotyping of patients with CF.

The present study has some limitations: (1) While the in silico character of the study represents an inherent limitation, the results are based on data acquired from actual patients’ CT scans and breathing patterns. Therefore, the maximum generation of airways in simulations is limited to the resolution of CT scans. FRI has been validated in adult patients with mild asthma who underwent CT and single-photon emission computed tomography (SPECT)/CT at their functional residual capacity and their total lung capacity [29]. In that study, CFD simulations of the tracer deposition administered with a nebulizer showed a good agreement (less than 3% of average variation) with CT and SPECT/CT in airflow distribution and hot spot detection. Further aerosol studies in asthmatic patients have yielded equivalent lung deposition results between FRI and scintigraphy studies for pressurized metered-dose inhalers (lung dose of 45% in scintigraphy studies vs. 46% with FRI for fluticasone/formoterol) [30,31] and dry powder inhalers (lung dose of %22 in scintigraphy studies vs. 29% with FRI for corticosteroids) [30,32]. (2) FRI uses the particle size distribution and the delivered dose data from in vitro experiments as input parameters. However, the Zirela nebulizer features a valved aerosol chamber that stores the aerosol produced by the nebulizer during the exhalation phase. Thus, a dense aerosol bolus accumulates in the aerosol chamber during the exhalation phase. Consequently, the amount of aerosol provided by the nebulizer is not constant for each breathing cycle. This time-dependence of the amount of available aerosol is not reflected in the FRI calculation, potentially leading to underestimating the amount of drug reaching the lungs in the actual patient.

## 4. Conclusions

FRI revealed a significant intrathoracic deposition of levofloxacin aerosols, which distributed preferentially to the lower lung lobes. The three-dimensional rendering of CF airways also detected structural differences between the airways of patients with mild and moderate CF, which possibly reflected in the difference seen in the central-to-peripheral deposition (C/P) ratio, suggesting that a later treatment at a more advanced disease might also lead to worse or less homogenous antibiotic deposition in the lungs. Thus, further studies evaluating severe ill patients and patients during exacerbations, where breathing patterns might be different, are highly interesting.

## Figures and Tables

**Table 1:** Patient characteristics.

	Mild CF	Moderate CF	*p*-Value
Number of patients	7	13	-
Sex (male/female)	5:2	9:4	0.919 *
Age (years)	23 (18–37)	27 (19–46)	0.248 ^#^
Height (cm)	172.5 (161–185)	169.8 (154–187)	0.523 ^#^
FEV_1_ (L)	3.6 (2.78–4.77)	2.2 (1.54–3.28)	<0.0001 ^#^
FEV_1_ (%pred)	91 (72–109)	57 (40–69)	<0.0001 ^#^

FEV_1_, Forced Expiratory Volume in 1 s; FEV_1_ (%pred), individual FEV_1_ compared to the FEV_1_ of the reference population. * Chi-square test for sex differences; ^#^ ANOVA test for age, height, FEV_1_, and FEV_1_ (%pred) comparisons. The mean and the range are shown.

## Data Availability

Data will be available upon reasonable request.

## Supplementary Materials

The following are available online at https://www.mdpi.com/article/10.3390/pharmaceutics13122051/s1, Supplemental Method 1: Description of the levofloxacin detection method, Table S1: Patients’ characteristics corresponding with the retrospective high-resolution CT scans used to reconstruct the 3D models of the airways, Table S2: Averaged breathing patterns and FEV_1_ (%pred) from patients with mild and moderate CF, Figure S1: Flow rate over time of the “Regular” breathing pattern and mild and moderate CF breathing patterns, Figure S2: Regional levofloxacin deposition in the moderate and mild CF lungs.
