# Supplementary material for: Deposition of Inhaled Levofloxacin in Cystic Fibrosis Lungs Assessed by Functional Respiratory Imaging
Source: Pharmaceutics. 2021 Dec 1;13(12):2051. doi: 10.3390/pharmaceutics13122051 (PMC8708197; doi:10.3390/pharmaceutics13122051)
Supplement: Supplementary file 1 [file pharmaceutics-13-02051-s001.zip › pharmaceutics-1414533-supplementary-done.pdf]

pharmaceutics

Supplementary Materials: Deposition of Inhaled Levofloxacin in Cystic Fibrosis Lungs Assessed by Functional Respiratory Imaging

Carsten Schwarz, Claudio Procaccianti, Benjamin Mignot, Hosein Sadafi, Nicolas Schwenck, Xabier Murgia, Federico Bianco

Supplemental Method 1. Levofloxacin was extracted from the collection filters and rinsed from the next generation impactor stages using an extraction solvent with an internal standard consisting of 100 µg/mL L-Tryptophan in Methanol/Water (80:20, *v/v*). A liquid chromatograph equipped with a Waters PDA 996 or UV 2487 detector coupled to a data handling system was used to determine levofloxacin concentrations.

Column: Synergy POLAR-RP 4µ, 80A 150 × 3.0 mm (Phenomenex)
Column temperature: 30 °C
Sample temperature: 5 °C
Flow Rate: 1 mL/min
Injection volume: 5 µL
Detector wavelength: 260 nm
Mobile Phase A: 20 mM Potassium Phosphate buffer pH 2.5
Mobile Phase B: Methanol
Composition: mobile Phase A / mobile Phase B = 65/35 (*v/v*)
Run time: 6 min
Rinse Solution: Methanol/Water = 50/50 (*v/v*)
Retention time LEVO: ~4.1 min
Retention time L-Tryptophan: ~1.5 min

Table S1. Patients' characteristics corresponding with the retrospective high-resolution computed tomography scans used to reconstruct the 3D models of the airways. Data were retrieved from the FLUIDDA database (informed consent was obtained from each patient, and ethical approval was granted by the Ethics Committee of the University Hospital in Antwerp, Belgium; file number: B300201731264; approval date 25/08/2017).

Subject	Age (yr)	Gender	Height (cm)	FEV_1 (L)	FEV_1 (% pred)	CF Stage
1	23	M	175.5	2.79	62	Moderate
2	26	M	169.5	2.25	54	Moderate
3	19	M	186.5	3.28	63	Moderate
4	33	F	160	1.57	51	Moderate
5	46	M	171	1.73	47	Moderate
6	25	M	168	2.35	58	Moderate
7	20	F	168	2.24	63	Moderate
8	22	F	154	1.54	53	Moderate
9	32	M	166	1.84	49	Moderate
10	24	M	186	1.94	40	Moderate
11	21	F	158.5	2.10	67	Moderate
12	36	M	176	2.80	69	Moderate
13	25	M	168	2.63	64	Moderate
14	22	F	161	2.78	96	Mild
15	22	M	178	4.77	107	Mild

16	21	F	174	3.42	93	Mild
17	18	M	169	4.42	109	Mild
18	37	M	176	3.02	72	Mild
19	19	M	165	3.04	78	Mild
20	22	M	185	3.94	82	Mild

M, male; F, female; FEV_1, forced expiratory volume in one second; FEV_1 (% pred), FEV_1 compared with the FEV_1 of the reference population; CF, Cystic Fibrosis.

Table S2. Averaged breathing patterns and FEV_1(% pred) from patients with mild and moderate Cystic Fibrosis. Data were retrieved from patients recruited from the CF clinics of the Hospital for Sick Children and St. Michael's Hospital in Toronto; all patients signed informed consent and the study was approved by the Research Ethics Boards (REB) of both institutions (REB number of the Hospital for Sick Children, 1000008378; REB number of the St. Michael's Hospital, 06-027). Briefly, patients were instructed to breathe through a laminar flow element (Fleisch Pneumotachograph Type I, Metabo SA, Epalinges, Switzerland) via mouthpiece and filter (PALL BB50T, Pall Corpotaion, Fribourg, Switzerland). The resulting pressure drop at the laminar flow element was recorded utilizing a PARI COMPAS Breath Monitor™ (Pari Pharma Gmbh, Starnberg, Germany).

CF Stage	**n**	**V_T (mL)**	**Rate (bpm)**	**Inspiratory phase (%)**	**FEV_1 (% pred)**
Moderate	4	608	23 ± 10	43 ± 3	80 ± 9
Mild	7	759	21 ± 9	47 ± 7	58 ± 5
All combined	11	703	22 ± 9	45 ± 27	72 ± 13

CF, Cystic Fibrosis; V_T, tidal volume, FEV_1 (% pred), forced expiratory volume in one second (FEV_1) compared with the FEV_1 of the reference population.

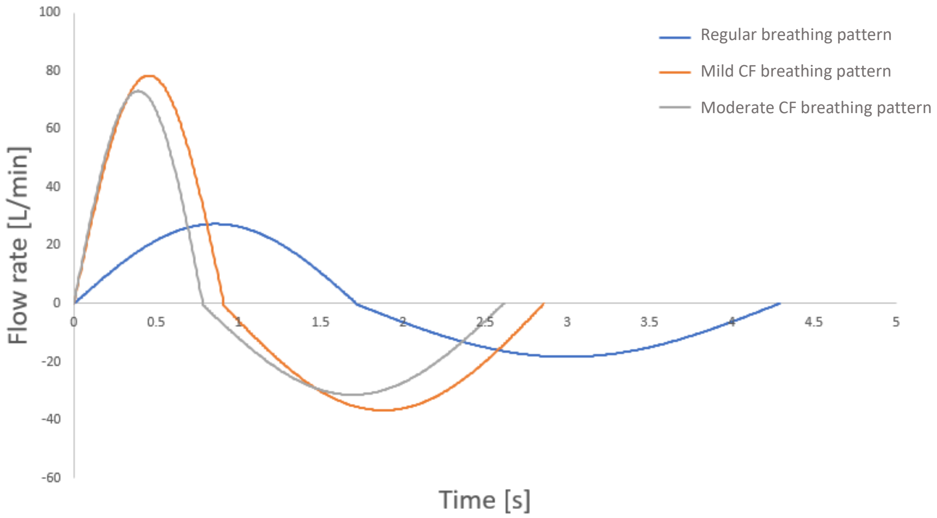

Figure S1: Flow rate over time of the "Regular" breathing pattern (obtained from the Pharmacopeia, blue line) and of the mild CF (orange line) and moderate CF (grey line) breathing patterns. Both CF breathing patterns, averaged from actual patients' data, show a steeper slope of the flow profile and a higher peak inspiratory flow.

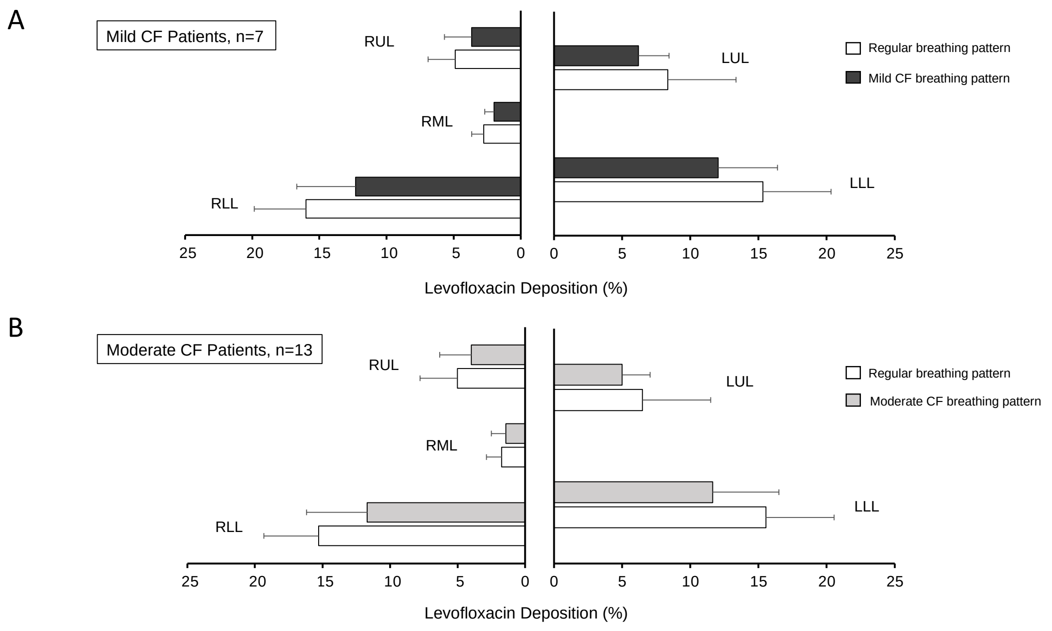

Figure S2: Regional levofloxacin deposition in the moderate and mild cystic fibrosis (CF) lungs. A) Mild breathing pattern (V_T of 0.759 L, rate of 21 cycles/min, I:E 1:2.3, mean flow rate of 46.5 L/min) compared with the regular breathing pattern (V_T of 0.5 L, rate of 14 cycles/min, I:E 1:1.5, mean flow rate of 17.5 L/min) in mild CF patients. B) Moderate breathing pattern (V_T of 0.608 L, rate of 23 cycles/min, I:E 1:2.14, mean flow rate of 50 L/min) compared with the regular breathing pattern in moderate CF lungs. RUL, right upper lobe; RML, right middle lobe; RLL, right lower lobe; LUL, left upper lobe; LLL, left lower lobe.
